# Association between testosterone levels and bone mineral density in females aged 40–60 years from NHANES 2011–2016

**DOI:** 10.1038/s41598-022-21008-7

**Published:** 2022-09-30

**Authors:** Han Zhang, Kun Ma, Run-Min Li, Jia-Ni Li, Shan-Feng Gao, Lin-Na Ma

**Affiliations:** 1 College of Traditional Chinese Medicine, Tianjin University of Traditional Chinese Medicine, Tianjin, China; 2 China Academy of Chinese Medical Sciences, No. 16, Nanxiao Street, Dongzhimen, Beijing, 100700 China; 3 First Clinical Medical College, Shandong University of Traditional Chinese Medicine, Jinan, China; 4 Xiyuan Hospital, China Academy of Chinese Medical Sciences, Beijing, China

**Keywords:** Endocrinology, Medical research

## Abstract

Growing evidence indicates that testosterone is a conspicuous marker for assessing male bone mineral density (BMD). However, research regarding testosterone levels and BMD is sparse and controversial for females. Hence, we aimed to investigate the association between testosterone levels and BMD among adult females aged 40–60 years in the United States. In this cross-sectional study, all participants were part of the National Health and Nutrition Examination Survey (2011–2016). A weighted general linear model was used to estimate the association between testosterone levels and lumbar BMD. Age, race, income level, education level, body mass index (BMI), blood urea nitrogen (BUN) level, serum uric acid (UA) level, serum calcium (Ca) level, serum phosphorus (P) level, the use of oral contraceptive pills, the use of hormone replacement therapy (HRT), smoking status, drinking status, and the use of corticosteroids were adjusted using a weighted multiple regression model. Subgroup analyses were performed using the same regression model. We included 2198 female participants in the study, and testosterone levels were positively associated with lumbar BMD after adjusting for all the covariates (*β* = 1.12, 95% CI 0.31, 1.93). In subgroup analyses, the associations in the fourth quartile of testosterone levels were stronger for the participants aged 40–50 years old (quartile 4, *β* = 42.92, 95% CI 7.53, 78.30 vs. quartile 1) and 50 to 60-year-old (quartile 4, *β* = 32.41, 95% CI 0.14, 64.69 vs. quartile 1). Similar results were found in other subgroups, including subgroups for race (Non-Hispanic Black, Other), income level (income ≤ 1.3, income > 3.5), education level (college or higher), BMI > 25 kg/m^2^, BUN levels ≤ 20 mg/dL, UA levels ≤ 6 mg/dL, Ca levels ≤ 10.1 mg/dL, P levels ≤ 5 mg/dL, drinking status, never smoker, never taking birth control pills, and HRT user. There was no interaction among the covariates in the association between lumbar BMD and testosterone levels (*P* for interaction > 0.05). In US adult females aged 40–60 years, the testosterone level was a positive predictor of the lumbar BMD after adjusting for covariates.

## Introduction

Along with the global social structure of population ageing, the trend of ageing causes an increase in the overall incidence and prevalence of osteoporosis^1^. A survey showed that more than half of women aged 60–70 years will suffer from postmenopausal osteoporosis^2^. Therefore, advancing the age threshold for research, observation and intervention is helpful to prevent and treat osteoporosis. Females aged 40–60 years are experiencing a special period in which females gradually transition from the childbearing period to menopause; in this period, ovarian function declines, hormone secretion changes^3^, the balance between bone absorption and bone formation is destroyed, bone loss accelerates, and the incidence of osteoporosis increases^4^. The decrease in testosterone may be closely related to this process^5^.

Osteoporosis is a disease of increased bone fragility owing to decreases in bone density and the destruction of bone microarchitecture. Osteoporosis is one of the most important causes of vertebral fractures in middle-aged and older people, seriously affecting patients’ quality of life and increasing socio-economic burden^2,6^. Lumbar BMD is a vital sign of bone quality, reflecting the degree of osteoporosis and predicting the risk of vertebral fracture. The lumbar spine is the site that is favoured for monitoring treatment, while the lumbar BMD test is one of the gold-standard techniques for the diagnosis of osteoporosis. The diagnostic method has been incorporated into several clinical guidelines^7,8^.

The relevant correlation analyses between testosterone levels and osteoporosis are limited to the male population^9,10^; few studies with the female population have been performed, with a lack of high-level evidence, and the study results are contradictory. Previous studies have found that testosterone is positively correlated with cortical BMD in females and is an independent predictor of BMD in healthy young females^11,12^. Postmenopausal females have lower testosterone and oestradiol (E_2_) levels than premenopausal females; hence, their BMD is reduced^13^. Clinical studies have shown that taking testosterone preparations can improve BMD in elderly women with osteoporosis^14,15^. However, another clinical study found that adding testosterone to oestrogen replacement therapy did not result in a significant increase in BMD^16,17^. A meta-analysis showed that testosterone substitution or therapy had no significant effect on BMD in females but sufficient evidence of safety was lacking^17^.

We designed this cross-sectional study based on the current state of research on the relationship between testosterone levels and BMD in females. We examined the associations between testosterone levels and lumbar BMD among US adult females aged 40–60 years using samples from a database of a multiracial population.

## Methods

### Data source and study population

Data for this study were obtained from the National Health and Nutrition Examination Survey (NHANES) (2011–2016). The National Center for Health Statistics (NCHS) adopted a multistage, complex clustered probability design to select a representative sample from United States civilians. All protocols were approved by the research ethics review board of the NCHS, and informed consent forms were obtained from all participants. The survey data and methodological details about the NHANES are available at http://www.cdc.gov/nchs/nhanes/.

### Study variables

The exposure variable was the testosterone level, which has strong androgenic and anabolic effects^9^. The isotope dilution liquid chromatography-tandem mass spectrometry (ID-LC–MS/MS) method was performed for routine quantitation of serum total testosterone^18^ based on the National Institute for Standards and Technology’s (NIST) reference method. This method was optimized for higher sample throughput and certified by the CDC Hormone Standardization Program (HoSt). Females with testosterone levels > 70 ng/dL are diagnosed as having hyperandrogenaemia. There are tumour and nontumor reasons for hyperandrogenemia^19^, including virilizing congenital adrenal hyperplasia, idiopathic hyperandrogenism, virilizing tumours, and polycystic ovary syndrome^19,20^. Consequently, we could not determine the reasons why the testosterone levels exceeded the normal range. To avoid the impact of these diseases on the results, we excluded participants with testosterone levels > 70 ng/dL^18^, focusing on the relationship between normal to low testosterone levels and lumbar BMD.

The outcome variable was lumbar BMD. It was quantified using dual-energy X-ray absorptiometry scans acquired on Hologic Discovery Model A densitometers^18^.

Variables thought to be confounders based on the literature and clinical judgement were included^10,21^. In this study, the covariates included demographic data (age, race, income level, education level, BMI), laboratory examinations (BUN, UA, Ca, P), and data from questionnaires (smoking status, having at least 12 alcohol drinks in the past year, ever taking birth control pills, ever using female hormones, ever taking prednisone or cortisone daily). The data acquisition process for testosterone levels, lumbar BMD, and the covariates can be found at the following URL: www.cdc.gov/nchs/nhanes/.

### Ethics statement

According to the Revised Declaration of Helsinki, the institutional review board (IRB) of the NCHS approved the use of NHANES datasets. Informed consent from all participants was obtained before data collection.

### Statistical analyses

Descriptive analysis was applied to all participants’ data. Categorical variables are expressed as proportions (%). As appropriate, continuous variables are expressed as the mean and standard deviation (SD) or median and interquartile range (IQR). Weighted multivariate linear regression models were used to evaluate the association between serum testosterone levels and lumbar BMD to assess differences in clinical characteristics.

*β* and 95% confidence intervals were calculated using multiple linear regression models for testosterone levels and lumbar BMD. Age, race, income level, education level, BMI, BUN levels, UA levels, P levels, Ca levels, oral contraceptive use, HRT use, smoking status, drinking status, and corticosteroid use were adjusted. A general linear model was used to study the association between testosterone levels and lumbar BMD. A *P* for trend was assessed to study trends of the association between testosterone levels and lumbar BMD among different testosterone levels. Participants were classified into age subgroups of ≥ 40 years and < 50 years old and ≥ 50 and ≤ 60 years old for the subgroup analyses. Participants were divided into two BMI groups using a cut-off value of 25 kg/m^2^. Participants were divided into two BUN level groups using a cut-off value of 20 mg/dL. Participants were divided into two UA level groups using a cut-off value of 6 mg/dL. Participants were divided into two Ca level groups using a cut-off value of 10.1 mg/dL. Participants were divided into two P level groups using a cut-off value of 5 mg/dL. These figures are critical values for normal and high values. A *P* for interaction > 0.05 indicates that no interaction was found. The analyses were performed with the statistical software package R (http://www.R-project.org, The R Foundation), and Free Statistics software version 1.4 (Beijing Free Kelin Medical Technology Co, Ltd.).

## Results

### Baseline characteristics of the study participants by categories of testosterone

**Figure 1:**
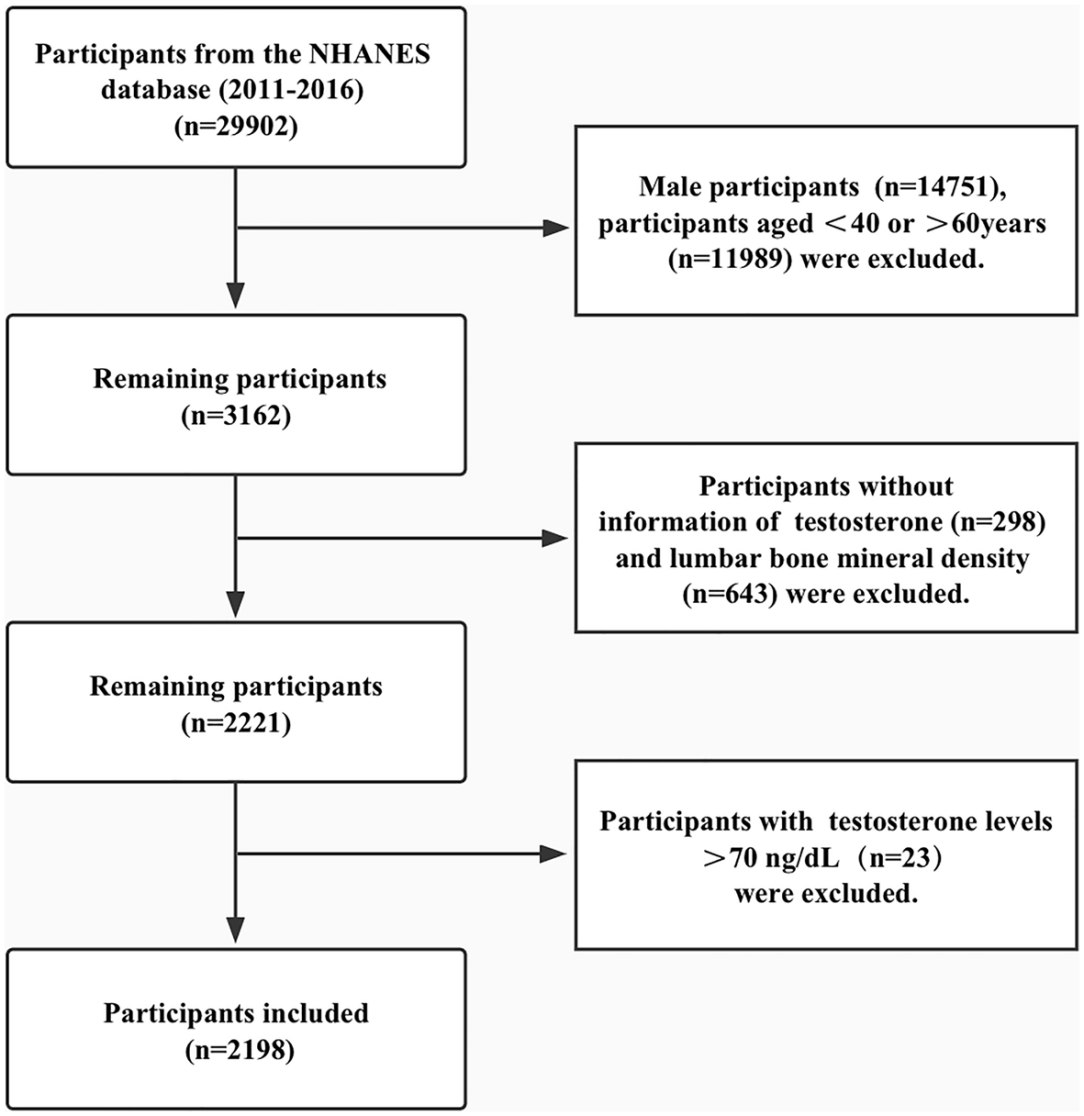
Flow chart of the screening process for the selection of eligible participants.

The population for the present analysis consisted of participants enrolled in 2011–2016. Among the 29,902 participants who underwent examinations, we excluded male participants (n = 14,751), those who were under the age of 40 years or over the age of 60 years (n = 11,989), those whose testosterone data (n = 298) or lumbar BMD data (n = 643) were missing and those with testosterone levels over 70 ng/dL (n = 23). Ultimately, 2198 participants were included in this study (Fig. 1).

Among the 2198 participants in the study, the average age was 49.1 years. The mean serum testosterone level was 18.9 ng/dL, and the mean lumbar BMD was 1017 mg/cm^2^. All variables were significantly different among persons classified into the different quartiles of testosterone. The lowest quartile of testosterone was ≥ 1.05 and < 12.15 ng/dL; the 2nd quartile was ≥ 12.19 and < 17.28 ng/dL; the 3rd quartile was ≥ 17.30 and < 23.25 ng/dL; and the highest quartile was ≥ 23.30 and < 68.20 ng/dL. Compared with participants in quartile 1, those in the other quartiles were younger, had higher lumbar BMD values, education levels, and BMI values, and had lower BUN and P levels. However, the drinking, hormone use rates, corticosteroid use rate, and oral contraceptive pills use rate were higher in quartile 1. The baseline characteristics of all participants are shown in Table 1.

**Table 1:** Description of the 2198 participants included in the present study.

Variable	All participants	Testosterone quartile (ng/dL)				*P*
		Q1 (1.05–12.15)	Q2 (12.19–17.28)	Q3 (17.30–23.25)	Q4 (23.30–68.20)	
**Participants (n)**	2198	550	547	547	554	
**Age (years)**	49.1 ± 5.6	50.4 ± 5.4	49.0 ± 5.5	48.9 ± 5.6	48.2 ± 5.7	< 0.001
**Race, n (%)**						0.001
Mexican American	335 (15.2)	97 (17.6)	99 (18.1)	75 (13.4)	64 (11.6)	
Non-Hispanic White	737 (33.5)	157 (28.5)	166 (30.3)	201 (36.7)	213 (38.4)	
Non-Hispanic Black	540 (24.6)	134 (24.4)	131 (23.9)	129 (23.6)	146 (26.4)	
Other	586 (26.7)	162 (29.5)	151 (27.6)	142 (26.0)	131 (23.6)	
**Income, n (%)**						0.883
Income ≤ 1.3	599 (29.7)	143 (28.7)	154 (30.6)	156 (30.9)	146 (28.7)	
1.3 < income ≤ 3.5	697 (34.6)	166 (33.3)	173 (34.4)	176 (34.9)	182(35.8)	
Income > 3.5	720 (35.7)	190 (38.1)	176 (35.0)	173 (34.3)	181 (35.6)	
**Education, n (%)**						0.297
High school or less	449 (20.4)	130 (23.6)	111 (20.3)	103 (18.8)	105 (19.0)	
Some college	455 (20.7)	116 (21.1)	103 (18.8)	114 (20.8)	122 (22.0)	
College or higher	1294 (58.9)	304 (55.3)	333(60.9)	330 (60.3)	327(59.0)	
**BMI (kg/m**^**2**^**)**	30.6 ± 7.6	30.1 ± 7.2	30.5 ± 7.3	30.6 ± 7.4	31.3 ± 8.3	0.053
**BUN (mg/dL)**	12.3 ± 4.6	13.2 ± 4.5	12.5 ± 4.7	12.1 ± 4.4	11.4 ± 4.9	< 0.001
**UA (mg/dL)**	4.8 ± 1.2	4.8 ± 1.3	4.8 ± 1.2	4.7 ± 1.2	4.8 ± 1.3	0.611
**Ca (mg/dL)**	9.3 ± 0.4	9.4 ± 0.4	9.3 ± 0.4	9.3 ± 0.3	9.4 ± 0.4	0.064
**P (mg/dL)**	3.8 ± 0.5	3.9 ± 0.6	3.8 ± 0.5	3.8 ± 0.5	3.7 ± 0.5	< 0.001
**Drinking status, n (%)**						0.062
No	775(35.3)	220 (40.0)	197(36.0)	179 (32.7)	179 (32.3)	
Yes	1249(56.8)	297 (54.0)	301 (55.0)	325 (59.4)	326 (58.8)	
Don’t know	174(7.9)	33(6.0)	49(9.0)	43(7.9)	49(8.8)	
**Smoking status, n (%)**						< 0.001
Never smoker	1422 (64.7)	372 (67.6)	377 (68.9)	350 (64.0)	323 (58.3)	
Former smoker	371(16.9)	102 (18.5)	88(16.1)	90(16.5)	91(16.4)	
Current smoker	405 (18.4)	76(13.8)	82 (15.0)	107(19.6)	140 (25.3)	
**OC use, n (%**)						0.439
Yes	1535 (69.8)	389 (70.7)	388 (70.9)	379 (69.3)	379 (68.4)	
No	486 (22.1)	128(23.3)	110 (22.1)	123 (22.5)	125 (22.6)	
Don’t know	177 (8.1)	33 (6.0)	49 (9.0)	45 (8.2)	50 (9.0)	
**HRT use, n (%**)						< 0.001
Yes	265 (12.1)	100 (18.2)	44 (8.0)	60 (11.0)	61 (11.0)	
No	1754 (79.8)	417(75.8)	453 (82.8)	443 (81.0)	441 (79.6)	
Don’t know	179 (8.1)	33 (6.0)	50 (9.1)	44 (8.0)	52 (9.4)	
**Corticosteroid use, n (%)**						0.193
Yes	57(3.9)	17 (4.7)	16 (4.6)	13 (3.6)	11 (2.9)	
No	777 (53.6)	197 (55.0)	167 (47.7)	198 (54.1)	215 (57.2)	
Don’t know	616 (42.5)	144 (40.2)	167 (47.7)	155 (42.3)	150 (39.9)	
**LBMD (mg/cm**^**2**^**)**	1017.0 ± 156.8	988.7 ± 154.6	1013.0 ± 159.3	1030.6 ± 157.5	1035.6 ± 152.1	< 0.001

Data presented are the mean ± SD or n (%). We categorized family income into the following 3 levels based on the family poverty income ratio: low income (≤ 1.3), medium income (> 1.3 to 3.5), and high income (> 3.5).

BMI, Body Mass Index; BUN, blood urea nitrogen; UA, serum uric acid; Ca, serum calcium; P, serum phosphorus. Drinking status, at least 12 alcohol drinks in the past one year; OCs, ever taking birth control pills; HRT, ever using female hormones; Corticosteroid use, ever taking prednisone or cortisone daily; LBMD, lumbar bone mineral density.

### Univariate linear regression analysis and the association between testosterone levels and lumbar BMD

Table 2 shows the results of the univariate linear regression analysis. We found that age, race, income level, education level, BMI, Ca levels, P levels, drinking status, smoking status, oral contraceptive use, and HRT use were significantly associated with lumbar BMD (*P* < 0.05). Among these, age, Ca levels, and P levels were negatively correlated with lumbar BMD. Income levels, BMI, and education levels were positively correlated with lumbar BMD (*P* < 0.05). BUN levels, UA levels, and corticosteroid use were not associated with lumbar BMD (*P* > 0.05). Compared with Mexican American women, Non-Hispanic White, Non-Hispanic Black, and women of other ethnicities aged 40–60 years had a higher lumbar BMD. Compared with never smokers, smokers had a lower lumbar BMD. Compared with drinkers, never drinkers had a higher lumbar BMD. Compared with participants who had never used HRT, those who did use HRT had a higher lumbar BMD. Compared with participants who never used oral contraceptive pills, those who did use oral contraceptive pills had a lower lumbar BMD.

**Table 2:** Univariate linear regression analyses.

Confounding factor category	*β* (95% CI)	*P* (t test)	*P* (F test)
**Age**	− 7.21 (− 8.34, − 6.08)	< 0.001	< 0.001
**Race: ref. Mexican American**			< 0.001
Non-Hispanic White	61.95 (42.35,81.55)	< 0.001	
Non-Hispanic Black	115.85 (95.16,136.54)	< 0.001	
Other	25.44 (5.06,45.81)	0.0140	
**Income: ref. income ≤ 1.3**			< 0.001
1.3 < income ≤ 3.5	19.15 (2.12,36.18)	0.028	
Income > 3.5	38.14 (21.24,55.05)	< 0.001	
**Education: ref. High school or less**			< 0.001
Some college	41.08 (20.86,61.31)	< 0.001	
College graduate or higher	61.7 (45.05,78.36)	< 0.001	
**BMI (kg/m**^**2**^**)**	1.63 (0.77,2.50)	< 0.001	< 0.001
**BUN (mg/dL)**	− 0.83 (− 2.24,0.58)	0.250	0.250
**UA (mg/dL)**	0.92 (− 4.39,6.23)	0.735	0.735
**Ca (mg/dL)**	− 40.16 (− 58.20, − 22.11)	< 0.001	< 0.001
**P (mg/dL)**	− 15.15 (− 27.24, − 3.05)	0.014	0.014
**Drinking status: ref. Yes**			< 0.001
No	29.32 (15.27,43.38)	< 0.001	< 0.001
**Smoking status: ref. Never smoker**			0.044
Former smoker	− 21.31 (− 39.23, − 3.39)	0.002	
Current smoker	− 2.24 (− 19.55,15.07)	0.800	
**OC use, ref. Yes**			< 0.001
No	− 41.82 (− 57.77, − 25.87)	< 0.001	
**HRT use, ref. No**			0.006
Yes	32.38 (12.10,52.66)	0.002	
**Corticosteroid use, ref. Yes**			0.684
No	18.95 (− 23.78,61.69)	0.384	

Data presented are the β and 95% confidence interval (CI), β (θ_1_, θ_2_). β is the effect size, θ_1_ > 0 indicates a positive correlation (P < 0.05), θ_2_ < 0 indicates a negative correlation (P < 0.05),

and θ_1_ < 0 < θ_2_ indicates no correlation (P > 0.05).

Table 3 shows the *β* and 95% CI for the association between testosterone levels and lumbar BMD. When analysed in continuous form, there was a significant positive correlation between testosterone levels and lumbar BMD, and this association was found in the unadjusted model (*β* = 1.56, 95% CI 0.89, 2.23), adjusted Model I (*β* = 0.94, 95% CI 0.17, 1.71) and adjusted Model II (*β* = 1.12, 95% CI 0.31, 1.93). When treated as a categorical variable, in the unadjusted model, as the level of testosterone increased, the BMD of the lumbar spine increased (*P* for trend < 0.001) in Q2 (*β* = 24.3, 95% CI 5.85, 42.75), Q3 (*β* = 41.92, 95% CI 23.47, 60.37), and Q4 (*β* = 46.9, 95% CI 28.51, 65.29). After adjustment for age, race and corticosteroid use, Q2 (*β* = 33.03, 95% CI 11.31, 54.75), Q3 (*β* = 37.65, 95% CI 16.18, 59.12), and Q4 (*β* = 35.90, 95% CI 14.38, 57.41), respectively, were assessed (*P* for trend < 0.001). There was an obvious positive correlation between testosterone levels and lumbar BMD in Q2, Q3, and Q4. After adjustment for age, race, income level, education level, BMI, BUN levels, UA levels, Ca levels, P levels, smoking status, drinking status, oral contraceptive pills use, HRT use, and corticosteroid use, a positive correlation still existed in Q2 (*β* = 23.45, 95% CI 0.69, 46.21), Q3 (*β* = 37.59, 95% CI 15.11, 60.08), and Q4 (*β* = 37.51, 95% CI 14.68, 60.35) (*P* for trend < 0.001).

**Table 3:** Association of testosterone levels with lumbar BMD.

	Nonadjusted Model	Adjusted Model I	Adjusted Model II
Testosterone (ng/dL)	1.56 (0.89,2.23)	0.94 (0.17,1.71)	1.12(0.31,1.93)
**Testosterone quartile**			
Q1 (1.05–12.15)	Reference	Reference	Reference
Q2 (12.19–17.28)	24.3 (5.85,42.75)	33.03 (11.31,54.75)	23.45 (0.69,46.21)
Q3 (17.30–23.25)	41.92 (23.47,60.37)	37.65 (16.18,59.12)	37.59(15.11,60.08)
Q4 (23.30–68.20)	46.9 (28.51,65.29)	35.90 (14.38,57.41)	37.51 (14.68,60.35)
P for trend	< 0.001	< 0.001	< 0.001

Data presented are the β and 95% CI. Model I was adjusted for age, race, and corticosteroid use.

Model II was adjusted for the variables in Model I + income level, education level, BMI, blood urea nitrogen level, serum uric acid level, serum calcium level, serum phosphorus level, drinking status, smoking status, ever taking birth control pills, and ever using female hormones. “P for trend” is mainly used to test whether there is a certain linear change trend between the change in the exposure variable of testosterone and the change in the outcome variable of lumbar BMD.

### Subgroup analyses of the association between testosterone levels and lumbar BMD

In the stratified analyses, the participants were divided into different levels according to covariates, and then the correlation strength between the covariates and BMD was analysed at each level. In regression, an interaction effect exists when the effect of an independent variable on a dependent variable change, depending on the value(s) of one or more other independent variables. To determine whether the association between testosterone levels and lumbar BMD was stable in different subgroups, we performed stratified analyses and interaction analyses (Table 4). The associations in the fourth quartile of testosterone levels were stronger for the participants aged 40–50 years old (quartile 4, *β* = 42.92, 95% CI 7.53, 78.30 vs. quartile 1). In addition, the associations between the fourth quartile of the 50 to 60-year-old subgroup and testosterone levels were also stronger (quartile 4, *β* = 32.41, 95% CI 0.14, 64.69 vs. quartile 1). Similar results were found in other subgroups, including subgroups for race (Non-Hispanic Black, Other), income level (income ≤ 1.3, income > 3.5), education level (college or higher), BMI > 25 kg/m^2^, BUN levels ≤ 20 mg/dL, UA levels ≤ 6 mg/dL, Ca levels ≤ 10.1 mg/dL, P levels ≤ 5 mg/dL, drinking status, never smoker, never taking birth control pills, and HRT user. There was no interaction among the covariates in the association between lumbar BMD and testosterone levels (*P* for interaction > 0.05).

**Table 4:** Subgroup analyses of the association between testosterone levels and lumbar BMD.

Confounding factor category	Testosterone quartile (ng/dL)				*P* for interaction
	Q1 (1.05–12.15)	Q2 (12.19–17.28)	Q3 (17.30–23.25)	Q4 (23.30–68.20)	
**Age (years)**					0.97
40–50	Reference	16.97 (− 18.96,52.90)	34.01(− 2.10,70.12)	42.92(7.53,78.30)	
51–60	Reference	22.26 (− 9.68,54.20)	29.8 (− 0.73,60.33)	32.41(0.14, 64.69)	
**Race, n (%)**					0.68
Mexican American	Reference	− 3.41 (− 54.97,61.79)	20.59 (− 42.67,83.86)	11.32 (− 57.13,79.78)	
Non-Hispanic White	Reference	− 5.49 (− 42.67,83.86)	− 0.49 (− 38.57,37.58)	17.57 (− 20.88,56.02)	
Non-Hispanic Black	Reference	53.26 (− 1.68108.20)	68.71 (15.60,121.81)	59.92 (7.14,112.70)	
Other	Reference	42.27 (− 1.45,86.00)	55.83 (10.93,100.72)	56.69 (10.02,103.37)	
**Income n (%)**					0.87
Income ≤ 1.3	Reference	5.37(− 41.39,52.13)	46.98 (1.50,92.45)	51.14 (3.14,99.14)	
1.3 < Income ≤ 3.5	Reference	25.93 (− 16.45,68.31)	31.13 (− 9.76,72.03)	24.42 (− 18.32,67.16)	
Income > 3.5	Reference	45.12 (7.10,83.14)	33.08 (− 5.90,72.06)	46.14(8.40,83.88)	
**Education, n (%)**					0.83
High school or less	Reference	29.56(− 27.47,86.59)	39.39 (− 16.35,95.13)	20.17 (− 39.33,79.66)	
Some college	Reference	20.48 (− 34.63,75.59)	21.06 (− 33.28,75.40)	9.44 (− 42.25,61.14)	
College or higher	Reference	27.74 (− 2.79,58.26)	36.43 (6.49,66.36)	50.02 (19.39,80.66)	
**BMI (kg/m**^**2**^**)**					0.96
≤ 25	Reference	5.03 (− 39.36,49.42)	24.34 (− 22.73,71.41)	18.11(− 26.86,63.08)	
> 25	Reference	29.67 (1.02,58.33)	37.48 (9.84,65.13)	43.69 (15.29,72.1)	
**BUN (mg/dL)**					0.69
≤ 20	Reference	22.27 (− 2.12,46.65)	33.33 (9.36,57.30)	20.03 (0.26,39.79)	
> 20	Reference	30.47 (− 147.82,202.76)	27.24 (− 129.52,94.00)	2.82 (− 170.05,175.68)	
**UA (mg/dL)**					0.94
≤ 6	Reference	20.34 (− 5.56,46.24)	32.93 (7.33,58.54)	31.66 (5.94,57.38)	
> 6	Reference	49.04 (− 16.30,114.39)	27.29 (− 35.01,89.59)	76.87 (8.16,145.58)	
**Ca (mg/dL)**					0.79
≤ 10.1	Reference	23.11 (− 1.25,47.47)	32.84 (8.96,56.72)	36.64 (12.24,61.05)	
> 10.1	Reference	23.10 (− 136.79,182.99)	− 6.61 (− 206.29,93.00)	54.76(− 95.24,204.76)	
**P (mg/dL)**					0.69
≤ 5	Reference	22.25 (− 1.92,46.41)	36.24 (12.43,60.04)	36.47 (12.40,60.53)	
> 5	Reference	− 115.11 (− 116.53,466.3)	− 34.39 (− 58.75,51.00)	− 85.15 (− 260.95,9.64)	
**Drinking status**				0.49	
No	Reference	11.36 (− 27.43,50.15)	43.00 (4.30,81.70)	43.04 (2.11,83.98)	
Yes	Reference	34.88 (4.23,65.54)	34.11 (4.18,64.05)	38.99 (9.00,68.98)	
**Smoking status**					0.89
Never smoker	Reference	30.73 (0.98,60.48)	28.59 (− 1.64,58.83)	40.01 (10.22,71.79)	
Former smoker	Reference	26.45 (− 27.99,80.89)	52.77 (0.35,105.19)	37(− 18.99,93.00)	
Current smoker	Reference	− 12.72 (− 78.64,53.21)	30.24 (29.65,90.13)	24.13 (− 33.66,81.92)	
**OC use**					0.92
No	Reference	28.71 (2.06,55.36)	39.87 (13.18,66.56)	43.38 (16.69,70.07)	
Yes	Reference	15.49 (− 40.11,71.08)	16.37 (− 33.24,66.98)	23.49 (− 30.74,77.73)	
**HRT use**					0.73
No	Reference	16.71 (− 55.21,88.62)	45.18 (− 18.10,108.46)	61.19 (− 0.99,123.38)	
Yes	Reference	26.53 (0.84,52.23)	36.59 (10.99,62.20)	36.34 (10.29,62.38)	
**Corticosteroid use**					0.56
No	Reference	31.22 (− 1.22,63.65)	38.30 (7.60,69.00)	25.48 (− 5.78,56.74)	
Yes	Reference	29.14 (− 108.83,167.11)	31.94 (− 112.07,175.9)	44.44 (− 92.90,181.79)	

Adjusted for age, race, BMI, education level, income level, blood urea nitrogen levels, serum uric acid levels, serum calcium levels, serum phosphorus levels, smoking status, drinking status, ever taking birth control pills, ever using female hormones, and ever using corticosteroids. A *P* for interaction > 0.05 represents no interaction.

## Discussion

In this nationally representative cross-sectional study, we combined data from the NHANES 2011–2016, and a total of 2198 females aged 40–60 years were included. In both univariate and multivariate linear regression analysis, our study indicated that testosterone levels were positively associated with lumbar BMD. The positive correlation between testosterone levels and lumbar BMD was still significant after adjusting for all the covariates and remained stable across subgroups. There was no interaction among the covariates in the association between lumbar BMD and testosterone levels.

According to the literature, it is basically clear that testosterone promotes BMD in males^22,23^, and the results from studies with females are controversial. On the one hand, some studies have found that testosterone levels in women are not related to BMD. A cohort study from California observed 457 females with a mean age of 72.1 years and found that testosterone levels were unrelated to BMD^24^. In another study, lumbar BMD and sex hormone levels were collected from 16 postmenopausal women. After statistical analysis, it was found that there was no correlation between testosterone levels and lumbar BMD. On the other hand, most studies are consistent with our results. A study from Athens suggested that higher testosterone levels may benefit the maintenance of female bone mass and that women with higher testosterone levels may be protected from the development of osteoporosis and fracture risk later in life^25^. These conflicting findings may be attributed to the heterogeneity among these studies, including differences in participant selection, study size, study design, and controlled confounders.

The current research focused on postmenopausal osteoporosis. However, the positive correlation between testosterone levels and BMD is not limited to menopausal females; females who are still menstruating may have relative deficiencies in testosterone, with reduced bone densities as a consequence. Bone density begins to decline in women before menopause^26^. The period of 40–60 years of age is the critical period from the beginning of bone mass reduction to the development of osteoporosis, and large sample clinical data analysis and cross-sectional studies are lacking for this period. Based on previous literature, our study fully considered confounding factors and strictly limited the age of the included population, making the conclusion reliable and filling in the gaps of current research from different perspectives. The mechanism by which testosterone increases BMD is unclear, and several possibilities have been proposed from the following aspects.

In cell culture studies, testosterone is sensitive to osteoblasts and osteoclasts^9^, and testosterone was shown to regulate osteoclast formation and survival associated with the RANKL pathway^27–29^. This suggests that testosterone or the nonaromatizable androgen dihydrotestosterone (DHT) acts directly on osteoclast progenitors and mature osteoclasts to inhibit osteoclastogenesis and promote osteoclast apoptosis^30^. Testosterone and DHT also regulate osteoclast activity and reduce bone turnover by inhibiting the production of PGE2 stimulated by parathyroid hormone and IL-1^31^. Another study showed that testosterone signalling through the androgen receptor (AR) expressed in cells of the mesenchymal lineage mediates the protective effects of testosterone on cancellous bone, indirectly decreasing osteoclast numbers and restraining bone resorption in this compartment^32^. Multiple studies have shown that isolated B lymphocytes can act as osteoclast progenitors^33–36^. Testosterone can inhibit the production of B cells and prevent bone loss^37–39^.

The relationship between oestrogen levels and BMD has been widely studied and confirmed^9,21,24,40^; oestrogen is widely used to prevent and treat osteoporosis. According to our univariate linear regression analysis, we found that female hormone users had higher BMDs. In the ovaries, testosterone is in part aromatized in granulosa cells by cytochrome P450aro (CYP19A1) to form E_2_^41^. A study found that with increasing age, the fraction of testosterone converted into E_2_ in the circulation increases^42^. Testosterone converted into E_2_ can not only enhance bone density and prevent osteoporosis but also improve menopausal symptoms caused by oestrogen deficiency.

However, the evidence for a positive effect of testosterone on bone health in women is contradictory^11,13,43,44^. Consequently, we used a large nationally representative sample with participants aged 40–60 years among the US female population, which increased the statistical strength to provide a more reliable result. The current findings have ideal generalizability. It is helpful for clinicians to identify groups at high risk for osteoporosis. There are also some limitations in our study. First, self-reported confounders might be susceptible to self-report bias. Second, limited by the cross-sectional study design, this study had less power regarding the determination of causal relationships between testosterone levels and lumbar BMD. Third, since the study was conducted in a population of middle-aged and elderly participants, the results may not be applicable to other age groups. Fourth, although we controlled for a broad range of lifestyle and health-related factors, correcting for possible confounders remained challenging. As we used questionnaires to estimate health status, residual confounding cannot be excluded. Fifth, we did not include E_2_ levels as a covariate because we were unable to determine whether the participants had their serum tested during the follicular phase; hence, the E_2_ value is unstable, and cannot accurately analyse the relationship between testosterone and E_2_, so the study could not look for an interaction between E_2_ and testosterone. Finally, due to the limited data, we only analysed the relationship between testosterone levels and lumbar BMD and did not further analyse the effects of androstenedione levels or free testosterone levels on lumbar BMD.

## Conclusions

In this cross-sectional study, we found a positive correlation between testosterone levels and lumbar BMD in American females aged 40–60 years. These findings will further deepen our understanding of the pathophysiology of osteoporosis in middle-aged and elderly women, and such a conclusion warrants further prospective studies with intervention trials.

## Data Availability

The data described in the manuscript, code book, and analytic code are available from the corresponding author upon request.

## Abbreviations

NHANES: The National Health and Nutrition Examination Survey
BMD: Bone mineral density
BMI: Body Mass Index
BUN: Blood urea nitrogen
UA: Uric acid
Ca: Calcium
P: Phosphorus
HRT: Hormone replacement therapy
E_2_: Oestradiol
NCHS: National Center for Health Statistics
ID-LC‒MS/MS: Isotope dilution liquid chromatography-tandem mass spectrometry
NIST: National Institute for Standards and Technology
HoSt: Hormone Standardization Program
IRB: Institutional review board
SD: Standard deviationor
IQR: Interquartile range
DHT: Dihydrotestosterone
AR: Androgen receptor
